# Development and evaluation of two subunit vaccine candidates containing antigens of hepatitis E virus, rotavirus, and astrovirus

**DOI:** 10.1038/srep25735

**Published:** 2016-05-19

**Authors:** Ming Xia, Chao Wei, Leyi Wang, Dianjun Cao, Xiang-Jin Meng, Xi Jiang, Ming Tan

**Affiliations:** 1 Division of Infectious Diseases, Cincinnati Children’s Hospital Medical Center, Cincinnati, OH, USA; 2 Department of Biomedical Sciences and Pathobiology, College of Veterinary Medicine, Virginia Polytechnic Institute and State University, Blacksburg, Virginia, USA; 3 Department of Pediatrics, University of Cincinnati College of Medicine, Cincinnati, OH, USA

## Abstract

Hepatitis E virus (HEV), rotavirus (RV), and astrovirus (AstV) are important pathogens that transmit through a common fecal-oral route, causing hepatitis (HEV) and gastroenteritis (RV and AstV) respectively in humans. In this study, we developed and evaluated two subunit vaccine candidates that consisted of the same protruding or spike protein antigens of the three viruses in two formats, a fusion of the three antigens into one molecule (fused vaccine) vs. a mixture of the three free antigens together (mixed vaccine). Both vaccines were easily made via *E. coli* expression system. Mouse immunization experiments showed that the fused vaccine elicited significantly higher antibody responses against the three viral antigens than those induced by the mixed vaccine. In addition, the mouse post-immune antisera of the fused vaccine revealed significantly higher neutralizing titers against HEV infection in cell culture, as well as significantly higher 50% blocking titers (BT_50_) against RV VP8-HBGA receptor interactions than those of the post-immune antisera after immunization of the mixed vaccine. Thus, the fused vaccine is a promising trivalent vaccine candidate against HEV, RV, and AstV, which is worth for further development.

Hepatitis E virus (HEV) of the family *Hepeviridae*^1^ is an important viral pathogen causing enterically-transmitted non-A, non-B viral hepatitis^2^. Each year HEVs cause approximately 20 million infections globally, resulting in around 3 million acute cases of hepatitis E and claiming 56,600 lives^3^. Rotavirus (RV) and astrovirus (AstV), members of the families of *Reoviridae* and *Astroviridae,* respectively, are common causative agents of gastroenteritis in humans^4^,^5^. RV infection causes severe diarrhea and dehydration among infants and young children^6^. A worldwide evaluation in 2008 showed that RV infection led to approximately 453,000 deaths in young children, accounting for 37% of deaths caused by diarrhea and 5% of all deaths in children younger than 5 years^5^. AstV is another leading causative agent of gastroenteritis in children under the age of 2 years, immunocompromised people, and the elderly^4^,^7^,^8^. AstVs are responsible for about 10% of sporadic nonbacterial diarrhea in children, with approximately 3.9 million cases of AstV gastroenteritis each year in the USA alone^9^. A seroprevalence study showed that 90% children in the USA have antibody reactive to human AstV-1 by the age of nine, suggesting that AstVs are highly prevalent in human populations^4^. Recent studies revealed that human AstVs are also associated with encephalitis^10^,^11^,^12^. All HEVs, RVs and AstVs spread via common fecal-oral route and they are important threats to public health.

HEVs and AstVs share important structural similarities. They both are nonenveloped RNA viruses, covered by a protein capsid that is constituted by a single major structural protein, the viral protein 1 (VP1) for AstVs and ORF2 Cap protein for HEVs^13^,^14^. Both viral capsids are featured by a number of exterior protrusions that are formed by the dimeric protruding (P) domains of VP1 or Cap^15^,^16^. These P domains interact with host ligands or receptors, playing an important role in the initiate steps of viral life cycle. Although RV is structurally distinct from HEV and AstV, it also has exteriorly protruding spike proteins formed by RV VP4^17^. The distal portion of the spike protein is formed by the VP8 domain that is responsible for host ligand or receptor interaction^18^. Thus, the viral protruding/spike proteins of HEV, RV, and AstV are excellent targets for subunit vaccine development against these three enterically transmitted viruses.

Two RV vaccines, RotaTeq (Merck) and Rotarix (GlaxoSmithKline), have been introduced to many countries worldwide since 2006, resulting in a significant decline in RV illness and childhood diarrhea deaths^19^,^20^. However, the two vaccines appear not to show satisfactory protection efficacy in developing countries^21^,^22^,^23^ and they remain expensive, making a large scale administration in the developing countries difficult. In addition, these two modified live-attenuated vaccines (MLVs) increase the risk of intussusception^24^,^25^,^26^,^27^,^28^,^29^,^30^. Thus, further improvement of the current vaccines and development of a new generation of safer, lower cost, and more efficient vaccines are warranted. The only HEV vaccine is a non-replicating subunit vaccine^31^ based on a recombinant E2 particle (HEV 239) that is composed of the truncated P1 and P2 domains of HEV ORF2^32^,^33^. This HEV vaccine is currently available only in China, while a commercial HEV vaccine remains lacking in other nations. On the other hand, the relatively poor growth of AstV in cell culture limited the development of both live and inactivated AstV vaccines. Although recombinant antigen-based, non-replicating subunit vaccines have been studied^34^,^35^,^36^, there is currently no vaccine against AstV so far.

The traditional MLVs and inactivated vaccine strategies are associated with certain safety concerns due to an involvement of live infectious virions. In contrast, a non-replicating subunit vaccine based on recombinant technology is not involved in an infectious virus and thus is considered safer with lower manufacturing cost than a traditional MLV vaccine. Four subunit vaccines have been commercially available in the USA, including Recombinvax (Merk) and Energix-B (GlaxoSmithKline) against hepatitis B virus, as well as Gardasil (Merk) and Cervarix (GlaxoSmithKline) against human papillomavirus. Together with the subunit HEV vaccine in China, these successful examples have endorsed the effectiveness of the subunit vaccine approach against various infectious agents.

The non-replicating subunit vaccines have a common shortcoming of relative low immunogenicity, particularly those based on small antigens with low valence. To this end, we have established a simple technology that can turn small antigens into large recombinant complexes for enhanced immunogenicity^37^,^38^. This is achieved by fusion of dimeric and/or oligomeric viral antigens together, while a monomeric antigen can also be presented by such complexes. In the present study, we fused the dimeric protruding (P) proteins of AstV and HEV together and then linked with the monomeric VP8 domain of RV spike protein (VP4), making a trivalent vaccine against the three viruses. We found that the fused vaccine formed dimers and elicited significantly higher immune responses to the three viral antigens than those induced by a mixture of the three individual free proteins (mixed vaccine). Our results indicated that the fused vaccine is a promising trivalent vaccine candidate against HEV, RV, and AstV.

## Results

### Expression and characterization of the fused and the mixed vaccines

**Figure 1:**
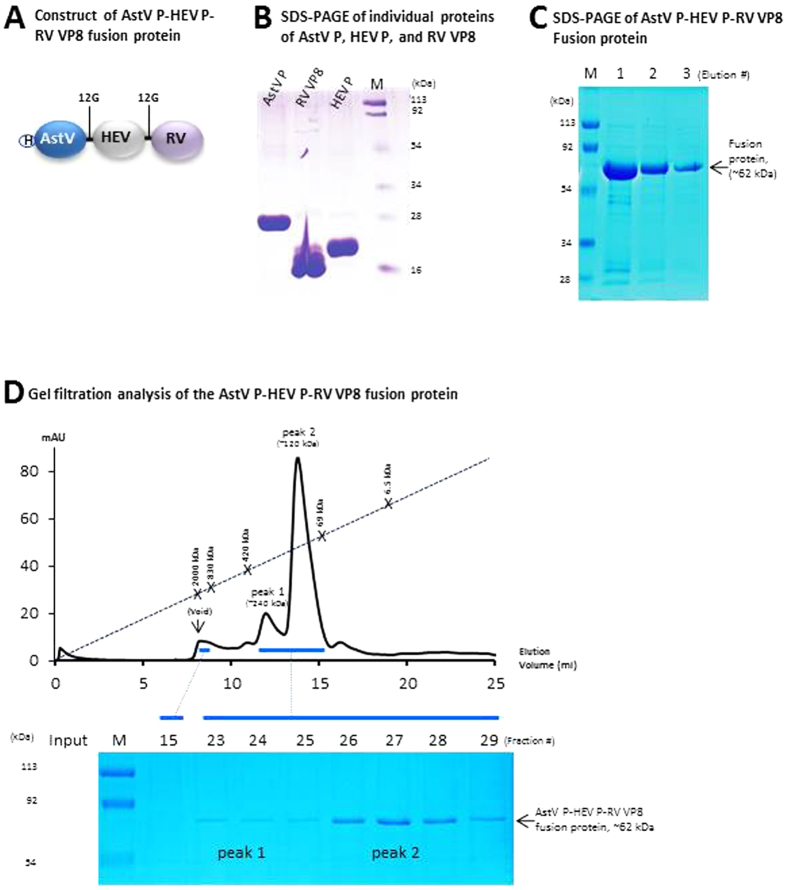
Production and characterization of the fused and mixed vaccines. (**A**) Schematic diagram of the fused vaccine (AstV P-HEV P-RV VP8) containing the dimeric P domains of astrovirus (AstV) and hepatitis E virus (HEV), as well as the monomeric VP8 protein of rotavirus (RV). A linker consisting of 12 glycines (12G) was added between the two P domains, while another one was between the HEV P domain and RV VP8. A histidine x6 tag (H) was linked to the N-terminus of the fused vaccine. (**B**,**C**) SDS PAGE of the affinity column-purified individual AstV P domain (AstV P, ~26 kDa), HEV P domain (HEV P, ~18.6 kDa), and RV VP8 protein (RV VP8, ~17.8 kDa) (B), as well as three elutions (lane 1, 2, and 3) of the fused vaccine (~62 kDa) consisting of the three viral antigens (**C**). (**D**) The elution curve of a gel filtration chromatography of the fused vaccine through the size-exclusion column Superdex 200 (10/300 GL, GE Healthcare Life Sciences). The gel filtration columns were calibrated by the Gel Filtration Calibration Kit (GE Healthcare Life Sciences) and the purified recombinant P particles^40^,^58^, small P particles^55^, and P dimers^53^ of norovirus (VA387). The elution positions of blue Dextran 2000 (~2000 kDa, void), P particle (~830 kDa), small P particle (~420 kDa), P dimer (~69 kDa), and aprotinin (~6.5 kDa) were indicated. The proteins of the two major peaks of the gel-filtrations were analyzed by SDS-PAGE that is shown below the elution curves with indications of peak 1 (fractions 23 and 24) and peak 2 (fractions 26 to 28), respectively. The elution ranges of the fused vaccine are indicated by blue bars. M represents pre-stained protein markers.

The three individual viral antigens were produced with expected sizes of ~26 (AstV P), ~18.5 (HEV P), and 17.8 (RV VP8) kDa, respectively (Fig. 1B). The fusion protein of the three viral antigens (Fig. 1A), the fused vaccine, was produced as a stable, soluble protein of ~62 kDa at a yield of ~15 mg/liter of bacterial culture (Fig. 1C). Gel filtration chromatography showed that vast majority of the fused vaccine formed dimer with a molecular weight (MW) of ~120 kDa (Fig. 1D, peak 2, fractions 26 to 29), while a small portion of the fused vaccine formed tetramer with a MW of ~250 kDa (peak 1, fractions 23 and 24). The free P domain proteins of AstV and HEV formed dimers, respectively^15^,^16^, while free RV VP8 formed monomer, which has been shown previously^39^.

### The vaccines elicited different levels of IgG responses to their antigen components

**Figure 2:**
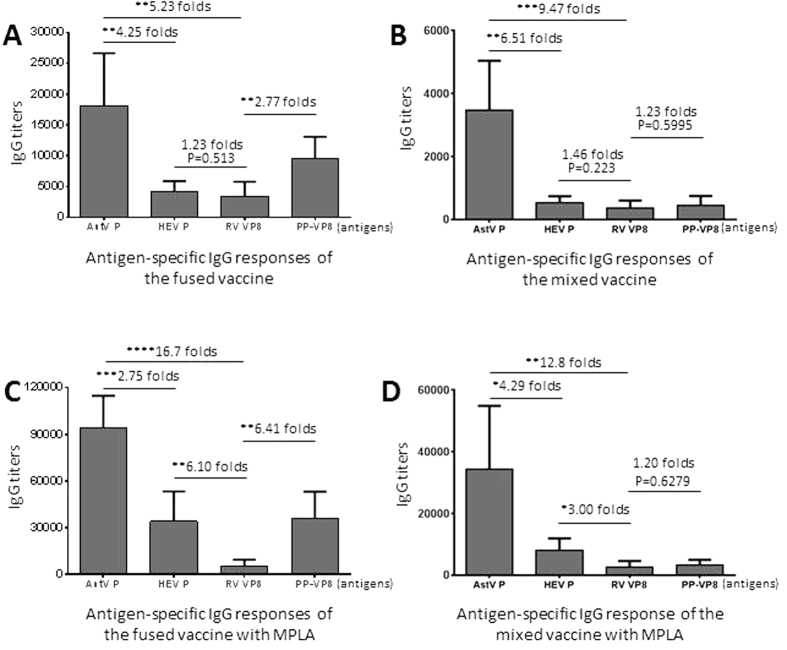
The three viral antigens of the fused and mixed vaccines elicited different levels of IgG responses. Mice (n = 6) were immunized with equal amount of the fused (**A**,**C**) and mixed (**B**,**D**) vaccines intranasally without (**A**,**B**) or with (**C**,**D**) MPLA adjuvant. The IgG titers specific to each individual viral antigen of astrovirus (AstV P), hepatitis E virus (HEV P), and rotavirus (RV VP8 and PP-VP8) after immunizations were measured using the purified P domains of AstV (AstV P) and HEV (HEV P), as well as free (RV VP8) and P particle-presented (PP-VP8) RV VP8 as capture antigens, respectively. (**A**,**B**) Comparisons of the IgG titers to the three viral antigens, respectively, after immunization of the fused (**A**) or mixed (**B**) vaccines without adjuvant. (**C**,**D**) Comparisons of the IgG titers after immunization of the fused (**C**) or mixed (**D**) vaccine with MPLA adjuvant. The differences between the data groups are indicated by folds, while their statistical differences are shown by *symbols (*P < 0.05, **P < 0.01, ***P < 0.001, ****P < 0.0001).

Mouse immunization experiments, including those with administration of the fused and the mixed vaccines, without or with MPLA adjuvant, respectively, showed the same trends of high and low IgG responses to the three viral antigens as determined by EIA using the purified individual viral proteins as capture antigens (Fig. 2, AstV P, HEV P, and RV VP8). The highest IgG titers was to the AstV P domain (AstV P), followed by those to the HEV P domain (HEV P) and then those to the RV VP8 protein. These high and low IgG responses may reflex the size and valence differences among the three viral antigens, among which the larger AstV P domain (26 kDa) and the HEV P domain (18.6 kDa) are dimeric^15^,^16^, while the smallest RV VP8 protein (17.8 kDa) is monomeric^39^. The higher RV VP8-specific IgG titers determined by the 24-meric P particle-presented VP8 (PP-VP8)^40^,^41^ than those by the monomeric free VP8 (RV VP8) indicated that the size and the valence of the capture antigen affected the outcome of the IgG titers (Fig. 2, compared column of RV VP8 with that of PP-VP8).

### The fused vaccine improved the IgG responses to their antigen components

**Figure 3:**
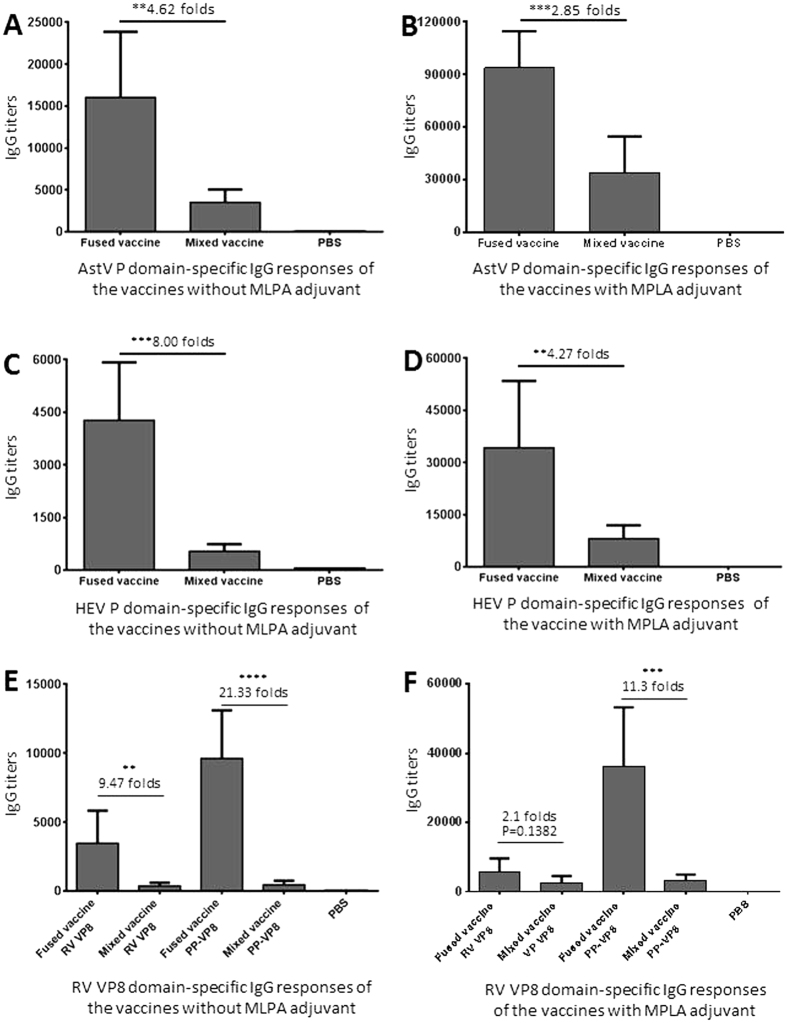
The fused vaccine increased IgG responses of the viral antigen components. Mice (n = 6) were immunized with the fused and the mixed vaccines intranasally without (**A**,** C** and **E**) or with (**B**, **D** and **F**) MPLA adjuvant. The resulting IgG titers specific to the individual viral antigens of astrovirus (AstV) P domain, hepatitis E virus (HEV) P domain, and rotavirus (RV) VP8, respectively, were measured using the purified P domains of AstV (AstV P) and HEV (HEV P), as well as free (RV VP8) and P particle-presented (PP-VP8) RV VP8 as capture antigens, respectively. Phosphate buffered saline (PBS) was administered to mice as negative control. (**A**, **C** and **E**) The fused and the mixed vaccine without adjuvant elicited IgG titers specific to AstV P domain (**A**), HEV P domain (**C**), and RV VP8 (**E**) were shown. (**B**,** D** and **F**) The fused and the mixed vaccine with adjuvant elicited IgG titers specific to AstV P domain (**B**), HEV P domain (**D**), and RV VP8 (**F**) were shown. The differences of the IgG titers between the two vaccines are indicated in folds, while their statistical differences are shown by *symbols (**P < 0.01, ***P < 0.001, ****P < 0.0001).

Comparisons of the IgG responses elicited by the fused and the mixed vaccines without adjuvant indicated that the fused vaccine induced significantly higher IgG responses to the three viral antigens than those elicited by the mixed vaccine, respectively (Fig. 3A, C and E, *Ps* < 0.01). The increase levels of the IgG titers were inversely correlated with their IgG responses with the highest increase to RV VP8 (>9.5 folds), followed by that to HEV P (8 folds) and then to AstV P domain (4.6 folds). Similar scenario was also seen, when the two vaccines were administered with MPLA adjuvant (Fig. 3B, D and F), but their increase levels became less compared with those without using MPLA adjuvant.

### MPLA adjuvant increased the IgG responses to all viral antigens

**Figure 4:**
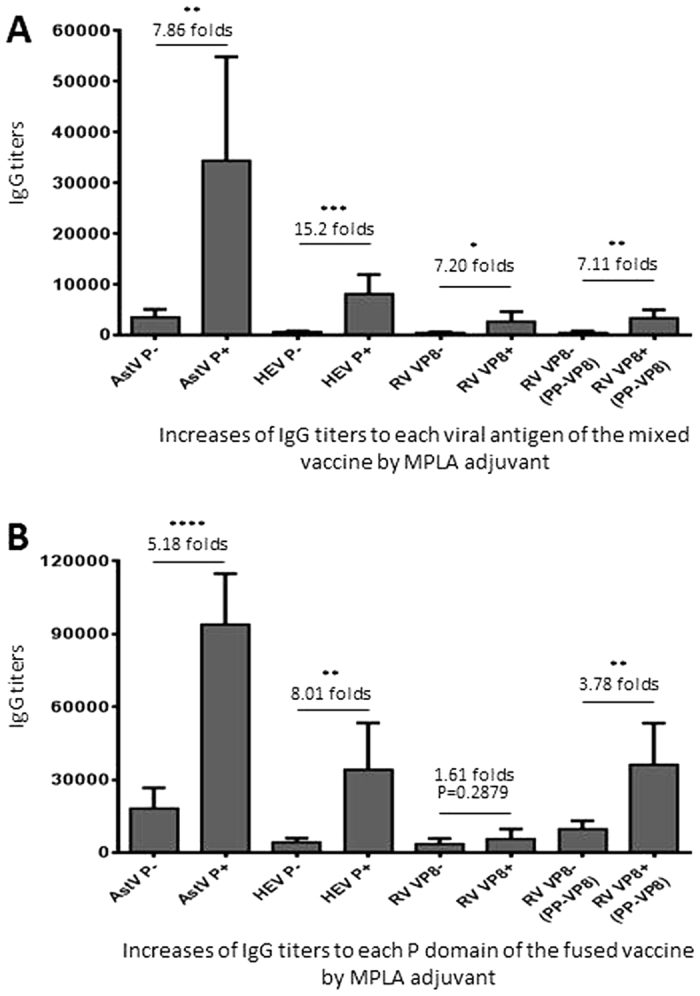
MPLA adjuvant increased the IgG responses of both fused and mixed vaccines. The viral antigen-specific IgG titers after immunization of with the mixed (**A**) and the fused (**B**) vaccines without (−) or with (+) MPLA adjuvant were shown and compared side-by-side. The IgG titers specific to each individual viral antigen of astrovirus P domain (AstV P), hepatitis E virus P domain (HEV P), and rotavirus VP8 (RV VP8) were measured using the purified P domains of AstV (AstV P) and HEV (HEV P), as well as free (RV VP8) and P particle-presented (PP-VP8) RV VP8 as capture antigens, respectively. The increased IgG titers in folds by MPLA adjuvant are indicated, while their statistical differences are shown by *symbols (*P < 0.05, **P < 0.01, ***P < 0.001, ****P < 0.0001).

Compared the viral antigen-specific IgG titers elicited by the two vaccines with and without MPLA showed that MPLA adjuvant significantly increased the IgG responses to all three viral antigen components (Fig. 4, *Ps* < 0.05). The highest titer increase was to HEV P domain (approximately 15 and 8 folds for the mixed and fused vaccines, respectively), followed by that to the AstV P domain (approximately 8 and 5 folds for the two vaccines, respectively), while RV VP8 domain showed the lowest increase in IgG responses by the MPLA adjuvant. It was noted that the fused vaccine generally elicited the higher IgG responses than those induced by the mixed vaccine with or without MPLA adjuvant, but the increase levels of IgG titers by MPLA adjuvant were higher for the mixed vaccine than those for the fused vaccine (Fig. 4, compared A with B).

### The post-immune antisera of the fused vaccine revealed increased neutralizing antibody titers against HEV

**Figure 5:**
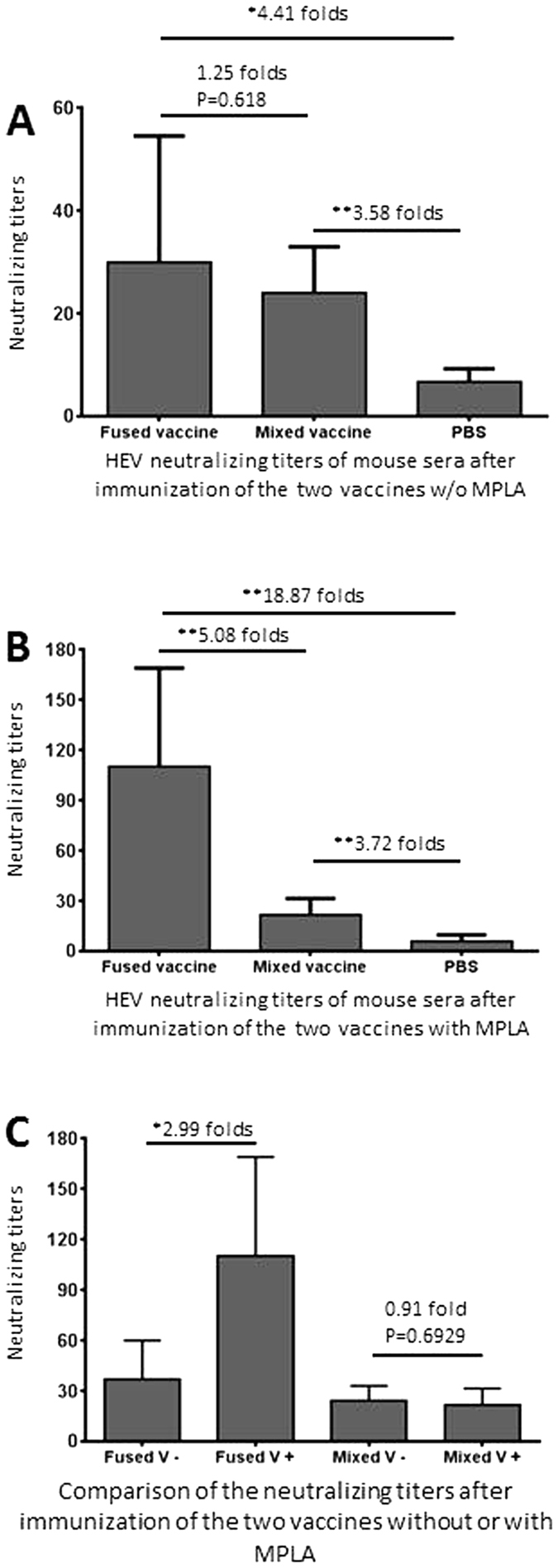
The post-immune antisera of the fused vaccine neutralized infection of hepatitis E virus (HEV)in cell culture. (**A**,**B**) Neutralization titers against HEV (Kernow P6) infection in HepG2/C3A cells were determined via fluorescent-focus assays using the post-immune antisera of the fused and mixed vaccines, respectively, without (**A**) or with (**B**) MPLA adjuvant. (**C**) The neutralizing titers of the mouse antisera after immunization of the fused and the mixed trivalent vaccines without (−) or with (+) MPLA adjuvant were compared. The differences of the neutralizing titers between the two vaccines are indicated in folds, while their statistical differences are shown by *symbols (*P < 0.05, **P < 0.01).

The post-immune mouse antisera of the fused and the mixed vaccines were determined for their neutralization titers against HEV (genotype 3 Kernow P6 strain) infection in HepG2/C3A cells (Fig. 5A,B). The neutralizing antibody titer of the post-immune antisera of the fused vaccine without adjuvant was higher than that of antisera after immunization of the mixed vaccine by 1.25 folds (Fig. 5A, *P* = 0.618). When vaccines were administered with MPLA adjuvant, the neutralizing antibody titer of the post-immune antisera elicited by the fused vaccine was 5.08 folds higher than that of the antisera after immunization of the mixed vaccine (Fig. 5B, *P* < 0.01). This result supported the notion that MPLA adjuvant significantly improved the immune responses (Fig. 4B) and neutralization titers (Fig. 5C, *P* < 0.05) of the fused vaccine.

### The fused trivalent vaccine-induced antisera revealed increased blocking titers against RV VP8-host ligand attachment

**Figure 6:**
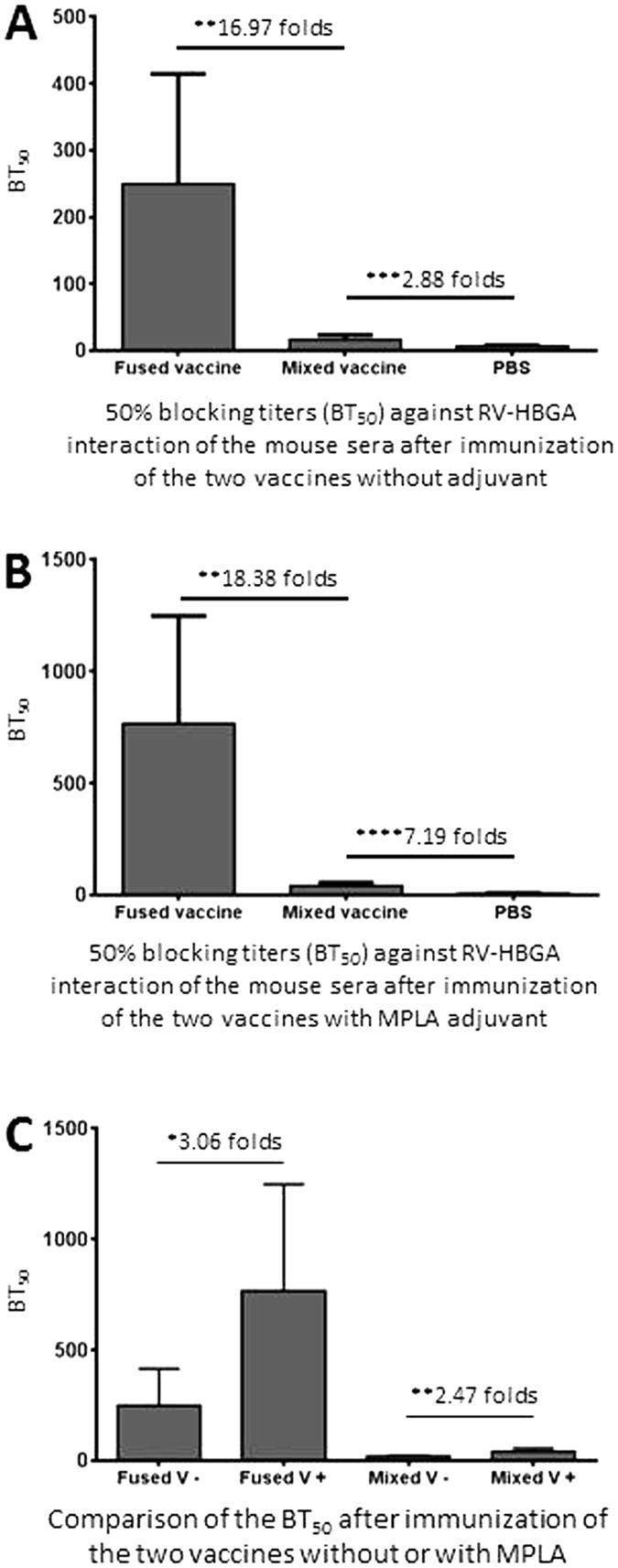
Serum blocking titers against attachment of rotavirus (RV) VP8 to its host ligands. The 50% blocking titers (BT_50_s) of the post-immune sera of the fused and mixed vaccines without (**A**) or with (**B**) MPLA adjuvant against the attachment of the P particle-presented RV VP8 (PP-VP8) to Lewis b (Le^b^) antigen-positive salivas were determined and compared. (**C**) The BT_50_s of the two vaccines without (−) and with (+) MPLA adjuvant were compared side-by-side. The differences of the neutralizing titers between the two vaccines are indicated in folds, while their statistical differences are shown by *symbols (*P < 0.05, **P < 0.01, ***P < 0.001, ****P < 0.0001).

Human histo-blood group antigens (HBGAs) were recently shown to be RV host ligands or receptors^39^,^42^,^43^,^44^ that play an important role in the RV infection in cell culture^44^ and disease development^45^,^46^. Since RV interacts with HBGAs via the VP8 domain of the spike protein, we measured the block titers of the mouse post-immune sera against RV VP8-host ligand attachment as a surrogate neutralization assay, the mouse antisera after immunization of fused and mixed vaccines were determined for their 50% blocking titers (BT_50_) against P particle-presented VP8^41^,^42^ attaching to human saliva containing Le^b^ HBGA (Fig. 6). The results showed that the post-immune sera of the fused vaccine exhibited significantly higher BT_50_s than those of the antisera after immunization with the mixed vaccine (Fig. 6A,B, *Ps* < 0.01). In addition, MPLA adjuvant increased the BT_50_s of the post-immune sera of both vaccines significantly (Fig. 6C, *Ps* < 0.05).

## Discussion

In this study, we developed and evaluated two subunit trivalent vaccines containing the neutralizing antigens of HEV, RV, and AstV. The vaccines were made via recombinant technology in two different formats, a trivalent fusion and a mixture of the three viral antigens, named fused and mixed vaccines, respectively. We evaluated the immune responses to the three individual antigens elicited by the two different vaccines to assess which vaccine is better for future development. Our data showed that the fused vaccine elicited significantly higher IgG responses to all three viral antigen components than those induced by the mixed vaccine. This observation was confirmed by the significantly higher neutralizing antibody titers against HEV in cell culture and the higher BT_50_s against attachment of RV VP8 to its HBGA host ligand by the post-immune mouse antisera of the fused vaccine than those of antisera after immunization of the mixed vaccine. Therefore, the fused vaccine is a better vaccine for future development.

The observed improved immune responses elicited by the fused vaccine than those by the mixed vaccine can be explained by their molecular sizes and valence that are two key factors affecting the immunogenicity of an antigen. The three viral antigens are ~26 (AstV P domain), ~18.7 (HEV P domain), and ~17.8 (RV VP8 domain) kDa, respectively. The individual antigens are smaller than that of the fused vaccine that is ~62 kDa. In fact, gel-filtration chromatography indicated that the vast majority of the fused vaccine formed dimers with a small fraction of tetramers, making the fused vaccine to be ~124 and ~248 kDa, respectively, much larger than the three individual viral antigens (17 to 26 kDa). Accordingly, the differences in the IgG responses of the three individual antigens of the two vaccines may also reflex their size and valence differences. The three viral antigens show a size order of AstV P domain > HEV P domain > RV VP8 domain, correlated with the order of their IgG responses. Although HEV P domain (18.6 kDa) and RV VP8 domain (17.8 kDa) are similar in sizes, the fact that AstV and HEV P domains form dimers^15^,^16^, but RV VP8 forms monomer^39^, increase their size differences. As a result, the AstV P dimer (~56 kDa) and the HEV P dimer (~37 kDa) are much larger than the RV VP8 monomer (~17.8 kDa).

In addition to immunogenicity, the size and valence differences of the capture antigens also affected the outcomes of the EIAs for IgG titer determination. For example, the RV VP8-specific IgG titers elicited by the fused vaccine were significantly higher using the 24-meric P particle-present VP8s as the capture antigen in the EIAs compared with those determined by the free monomeric VP8 (Fig. 2, compared A with C). Same trends were also seen for the mixed vaccine, but the differences were not statistically significant (Fig. 2, compared B with D), most likely due to the very low immunogenicity of the monomeric free RV VP8 in the mixed vaccine. Thus, the observed IgG titers of the vaccines should be the total outcomes of the vaccines with given antigens as both immunogens in the immunization and capture antigens in the EIAs.

This fused trivalent vaccine was modified from a previously made trivalent vaccine that consists of three dimeric P domains of HEV, AstV, and norovirus (NoV), named AstV P-HEV P-NoV P^47^, which was designed and constructed based on our newly developed technology^37^,^38^. The dimeric NoV P in the previous trivalent vaccine was replaced by the monomeric RV VP8, leading to the current fused trivalent vaccine, named AstV P-HEV P-RV VP8. The observed significantly improved immune response to the RV VP8 antigen of the fused vaccine compared with that of the mixed vaccine indicated that a monomeric antigen can be presented by a fused complex for enhanced immunogenicity. This example may be extended to other monomeric antigens for improved immune responses for future vaccine development.

MPLA was apparently an excellent adjuvant to significantly improve the immune responses of the fused vaccine via an internal immunization. In our study, even when a half amount of vaccine was used, MPLA adjuvant was able to increase the IgG titers to the three viral antigens of the fused vaccine for at least 7 folds compared with those without adjuvant (Fig. 4A). Since MPLA, a detoxified form of the endotoxin lipopolysaccharide, is a new generation of vaccine adjuvants to be used in human populations^48^,^49^, future assessment of this fused vaccine should be associated with this adjuvant.

## Methods

### Vaccine design

The fused vaccine was designed as a trivalent subunit vaccine, referred as AstV P-HEV P-RV VP8. It was a fusion protein that consists of the P domain of an avian AstV (GenBank AC#: NP 987088, residue 423-630)^16^, the P domain of a zoonotic genotype 3 HEV from a pig^50^ (GenBank AC#: DQ079627, residue 452 to 617, a part of the E2 protein)^15^,^51^, and the VP8 antigen of a human RV (P[8], GenBank AC#: VPXRWA, residue 65 to 223)^37^,^41^ (Fig. 1A). A short peptide containing four cysteines (CDCRGDCFC) was added to the C-terminus of the HEV P domain to stabilize the recombinant protein^37^. A hinge consisting of 12 glycines (12G) was added between the AstV P domain and the HEV P domain, as well as between the HEV P domain and the RV VP8 domain. A hisx6 tag was linked to the N-terminus of the fusion protein for purification purpose (Fig. 1A). The mixed vaccine was a mixture of equal amount of the three viral antigens.

### Expression constructs

The plasmid for expression of the AstV P-HEV P-RV VP8 fusion protein was based on the pQE30 vector (Qiagen, Germantown, MD) containing the coding sequences of AstV P, HEV P and RV VP8 domains. The related coding sequences were re-cloned from the previously made constructs of the GST-HEV P and GST-VP8^37^. The AstV P domain-encoding sequences were synthesized chemically via Genscript (Piscataway, NJ). A plasmid containing the pQE30 and AstV P-12G-HEV P coding sequences (designated as pQE30-AstV P-12G-HEV P) was first constructed as described previously^47^ using two primer pairs, P2083/P2104 (AstV P) and P2062/P2105 (HEV P) (Table 1). Other two primer pairs, P2083/P2142 (AstV P) and P2145/P2146 with BamHI/BsmBI and BsmBI/SalI sites, respectively (Table 1), were then used to amplify AstV P-12G-HEV P and RV VP8-encoding cDNA sequences, respectively, followed by enzyme digestion, ligation and cloning into the pQE30 vector (Fig. 1A)^52^. The expression constructs for production of Hisx6 tagged AstV P, HEV P and RV VP8 domains were made by cloning the corresponding P domain coding sequences into the pQE30 vector, respectively.

### Vaccines production and purification

Recombinant vaccines were expressed in *E. coli* (BL21, DE3) as described previously^37^,^53^. The hisx6-tagged fusion protein and the individual P domains and VP8 proteins were purified by TALON CellThru Resin (ClonTech) according to manufacturer’s instruction.

### SDS-PAGE and protein quantitation

Quality of the purified proteins were evaluated by SDS-PAGE using 10% separating gels. The vaccines were quantitated by SDS-PAGE using serially diluted bovine serum albumin (BSA, Bio-Rad) as standards on same gels^41^. The gels were stained with PageBlue Protein Staining Solution (ThermoFisher Scientific).

### Gel filtration

Gel filtration chromatography was performed to analyze the size distributions of the vaccines as described elsewhere^37^,^53^,^54^. A size exclusion column (Superdex 200, 10/300 GL, GE Healthcare Life Sciences) controlled by an AKTA Fast Performance Liquid Chromatography System (AKTA Pure 25L, GE Healthcare Life Sciences) was used. The column was calibrated using gel filtration calibration kits (GE Healthcare Life Sciences) and purified NoV P particles (~830 kDa)^54^, small P particles (~420 kDa)^55^, and P dimers (~69 kDa)^53^ as described previously. The protein identities corresponding to the peaks of the elusion curves were characterized by SDS-PAGE.

### Immunization of vaccines

Female mice (BALB/c, Harlan-Sprague-Dawley, Indianapolis, IN) at 3–4 weeks of age were divided into three groups (N = 6). Two groups were immunized with equal molar amount of the fused vaccine and the mixed vaccine, respectively, while the third group was immunized with phosphate buffered saline (PBS, pH 7.4) as negative control. For vaccines administration without adjuvant, a dose of 15 μg/mouse of the fused vaccine and the same 15 μg/mouse dose of the mixed vaccine consisting of 5 μg/mouse of each individual viral antigen were used to have roughly equal molar amounts of the fused and mixed vaccines. For vaccine immunization with monophosphoryl lipid A (MPLA, 5 μg/dose) adjuvant, a dose of 7.5 μg/mouse of the fused vaccine and the same of 7.5 μg/mouse dose of the mixed vaccine consisting of 2.5 μg/mouse of each individual viral antigen were used. Vaccines were administered three times intranasally with or without adjuvant in 2-week intervals as described previously^37^,^41^. Blood was collected by retro-orbital capillary plexus puncture before each immunization and two weeks after the final immunization. Sera were processed from blood via a standard protocol.

### IgG titer determination

The antibody IgG titers specific to each individual viral antigen of the post-immune mouse antisera were measured by enzyme immunoassay (EIA), as described previously^41^. Gel-filtration-purified P domain/spike proteins of AstV, HEV, and RV, as well as the P particle-presented RV VP8^41^ were used as antigens in the EIA. The viral antigens at 1 μg/ml were coated on 96-well microtiter plates and incubated with serially diluted mouse sera. Bound antibodies were detected by goat-anti-mouse secondary antibody-HRP conjugates (MP Biomedicals, Inc). Antibody titers were defined as the end-point dilutions with a cutoff signal intensity of 0.15.

### HEV neutralization assay

This was performed to measure HEV neutralization by post-immune mouse sera of the fused and mixed vaccines, in which the Kernow P6 HEV strain (genotype 3, kindly provided by Dr. S.U. Emerson, NIAID) and HepG2/C3A cells were used as described previously^56^,^57^. HEV infectious titers in focus forming units (FFU) were determined by a fluorescent-focus assay (FFA). ~50,000 HepG2/C3A cells/well were seeded in 96-well plates. The viruses (100 FFU/well) were mixed with the 2-fold serially diluted mouse sera for 2 hours at 37 °C and then added to the cells. After a 2-hour incubation, the inocula were discarded and replaced with maintenance medium. After further incubation for 5 days, the infected cells were fixed with 80% acetone and incubated with rabbit anti-HEV ORF2 antibody, washed with PBST (1xPBS with 0.2% tween-20), and then incubated with Alexa Fluor^®^ 488 Goat Anti-Rabbit IgG Antibody. The stained cells were visualized via a fluorescence microscope. The neutralization titers of the sera were defined as the highest serum dilution that can reduce at least 60% of infected cells compared with no serum controls.

### Serum blocking titers against attachment of RV VP8 to its host ligands

This procedure was adapted from the serum blocking assay of NoV-HBGA (histo-blood group antigen) interaction^37^ as a surrogate neutralization assay. Boiled and diluted (1:1000) human saliva samples with defined Lewis b (Le^b^) antigen, the host ligand of P[8] RV, were coated on microtiter plates. The P particle-presented RV VP8 (PP-VP8) at 0.625 μg/mL was pre-incubated with the post-immune mouse sera after immunization with fused or mixed vaccines at different dilutions before the PP-VP8 was added to the coated saliva samples. The 50% blocking titers (BT_50_s) were defined by the serum dilution that caused 50% blocking compared with the unblocked positive control.

### Statistical analysis

Statistical differences among data sets were calculated by softwares GraphPad Prism 6 (GraphPad Software, Inc) using an unpaired, non-parametric *t* test. *P*-values were set at 0.05 (*P* < 0.05) for significant difference, and 0.01 (P < 0.01) for highly significant difference.

### Ethics statement

This study was carried out in strict accordance with the recommendations in the Guide for the Care and Use of Laboratory Animals (23a) of the National Institutes of Health. The protocols were approved by the Institutional Animal Care and Use Committee (IACUC) of the Cincinnati Children’s Hospital Research Foundation (Animal Welfare Assurance no. A3108-01).

## Additional Information

**How to cite this article**: Xia, M. *et al.* Development and evaluation of two subunit vaccine candidates containing antigens of hepatitis E virus, rotavirus, and astrovirus. *Sci. Rep.* **6**, 25735; doi: 10.1038/srep25735 (2016).

## Figures and Tables

**Table 1:** Primers that were used to generate expression constructs of recombinant proteins.

Construct	Name	Sequence (5′ to 3′)^a^	Sense	Enzyme	Note
pQE30-AstVP-12G-HEV P	P2083	GCACGGATCCTCTATCTACCTGCCGCTGC	+	BamHI	AstV P domain
	P2104	ATATCGTCTCCTCCGCCTCCGCCTCCGCCTCCGCCTCCGCCTCCGCCGAACTGAACGGTACG	-	BsmBI	AstV P domain (contains sequences for 12 Glycines)
	P2062	TATTCGTCTCCCGGATCTCCGGCTCCATCTCGTCCGTTCTCTGTTC	+	BsmBI	HEV P domain
	P2105	GCACAAGCTTTTACGGGTAGTCAACGGTGTCTTC	-	HindIII	HEV P domain
pQE30-AstVP-12G-HEVP-12G-RV VP8	P2142	ATATCGTCTCCCGCCGCAAAAGCAATCGCCACGGCAATCGCACG	−	BsmBI	HEV P domain
	P2145	TATTCGTCTCCGGCGGAGGCGGAGGCGGAGGCGGAGGCGGAGGCGGATTAGATGGTCCTTATCAAC	+	BsmBI	RV VP8 domain (contains sequences for 12 Glycines)
	P2146	ATATGTCGACTTATAGACCGTTGTTAATATATTCATTACACTTAGACTCTTG	-	SaII	RV VP8 domain

^a^Restriction enzyme recognition sites are underlined.
